# Variabilidade Fenotípica em uma Família com Síndrome de Andersen-Tawil

**DOI:** 10.36660/abc.20240861

**Published:** 2026-01-26

**Authors:** Samuel Ulisses Chaves Nogueira do Nascimento, Jussara de Oliveira Pinheiro Duarte, Luiz Pereira de Magalhães, Alex Teixeira Guabiru, Adimeia Souza Santos, Enderson Cavalvanti, Victoria Bastos Rodrigues, Roque Aras

**Affiliations:** 1 Hospital Universitário Professor Edgard Santos Salvador BA Brasil Hospital Universitário Professor Edgard Santos, Salvador, BA – Brasil; 2 Universidade Federal da Bahia Salvador BA Brasil Universidade Federal da Bahia, Salvador, BA – Brasil

**Keywords:** Síndrome de Andersen, Paralisia, Arritmias Cardíacas, Canalopatias, Genética Humana

## Introdução

As canalopatias cardíacas são distúrbios genéticos causados por variantes patogênicas em genes que codificam canais iônicos, resultando em alterações na eletrofisiologia cardíaca e predisposição a arritmias potencialmente fatais. Dentre essas condições, a Síndrome do QT Longo (SQTL) se destaca como uma entidade clínica heterogênea, caracterizada pelo prolongamento do intervalo QT e risco aumentado de taquiarritmia ventricular e morte súbita.

Algumas formas hereditárias da SQTL ocorrem no contexto de síndromes genéticas, como a Síndrome de Andersen-Tawil (SAT), causada por variantes no gene *KCNJ2*, que codifica o canal de potássio Kir2.1. Além do QT longo e da predisposição a arritmias ventriculares, a SAT se manifesta com paralisia periódica e dismorfias craniofaciais, compondo uma tríade diagnóstica. Apesar do diagnóstico poder ser clínico, a confirmação molecular é essencial para a estratificação de risco, o aconselhamento genético e a diferenciação de outras condições com sobreposição fenotípica.^1^

Este relato descreve uma família com SAT associada à variante *KCNJ2:p.Thr75Met*, ressaltando a expressividade variável e um achado fenotípico incomum na filha do caso índice, a presença de anomalia de Ebstein, que não é associada à síndrome. A correlação entre achados clínicos, moleculares e funcionais enfatiza a relevância deste caso para a compreensão da SAT e suas manifestações atípicas.

## Descrição

O caso índice, um homem de 52 anos, foi encaminhado à nossa equipe por quadro de crises de paralisia periódica desde os 14 anos, caracterizadas por perda de movimentos voluntários nos membros inferiores, com duração de 3 a 5 dias. Aos 25 anos, as crises se intensificaram, afetando também os membros superiores e sendo desencadeadas por repouso pós-exercício, ingestão de carboidratos e corticosteroides. Relatava palpitações esporádicas associadas ao estresse. A irmã faleceu subitamente aos 37 anos, com possível envolvimento cardíaco relacionado à SAT, um irmão apresenta quadro de paralisias periódicas desencadeadas por exercício.

**Figura 1:**
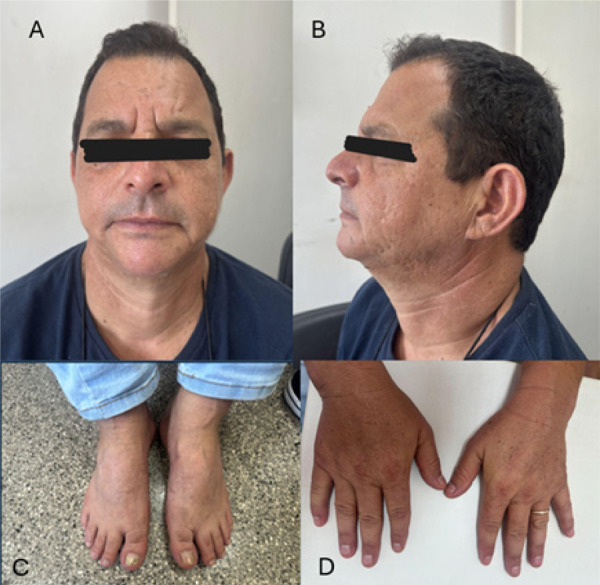
– Exame físico revelou micrognatia, orelhas de implantação baixa, fenda palpebral oblíqua para baixo, palato ogival, clinodactilia do quinto dedo bilateralmente e sindactilia dos segundo e terceiro pododáctilos.

**Figura 2:**
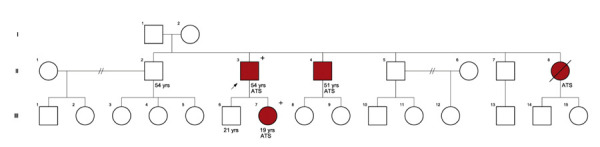
– Heredograma mostrando padrão autossômico dominante da Síndrome de Andersen-Tawil (SAT). Pacientes em vermelho possuem diagnóstico clínico de SAT. Os pacientes que realizaram teste genético e confirmaram são indicados com “+”. Os outros familiares ainda aguardam realizar teste genético, apesar de possuírem diagnóstico clínico.

O exame físico do caso índice revelou micrognatia, orelhas de implantação baixa, fenda palpebral oblíqua para baixo, palato ogival, clinodactilia do quinto dedo e sindactilia dos segundo e terceiro pododáctilos (Figura 1). O eletrocardiograma (ECG) mostrou intervalo QT prolongado, bradicardia sinusal, desvio do eixo do QRS para a esquerda e bloqueio incompleto de ramo direito. O Holter de 24 horas identificou bigeminismo ventricular; ecocardiograma confirmou fração de ejeção preservada (79%), sem alterações estruturais significativas. O sequenciamento completo do exoma identificou a variante c.224C>T (dbSNP: rs104894585), resultando em p.Thr75Met no gene *KCNJ2* (NM_000891.3) em heterozigose. A classificação como patogênica seguiu os critérios ACMG:^2^ predições in sílico (PP3), estudos de caso-controle (PS4), ensaios funcionais demonstrando perda de função do canal Kir2.1 (PS3), baixa frequência populacional (PM2), segregação familiar (PP1) e relatos prévios na literatura (PS1).^3-6^ O heredograma (Figura 2) confirmou o padrão de herança autossômico dominante.

Os filhos do paciente foram avaliados com o objetivo de investigar a presença da variante identificada no caso índice e possíveis manifestações clínicas associadas. A filha apresentava dismorfismos semelhantes aos do pai, mas não referia episódios de paralisia periódica. Seu eletrocardiograma revelou prolongamento acentuado do intervalo QT corrigido (QTc = 579 ms). O Holter de 24 horas evidenciou atividade ectópica ventricular significativa, com episódios de bigeminismo, extrassístoles ventriculares predominantemente polimórficas e taquicardias ventriculares não sustentadas, algumas com morfologia polimórfica. O teste ergométrico confirmou a presença de arritmias ventriculares polimórficas desencadeadas pelo esforço físico. O ecocardiograma transtorácico mostrou achados compatíveis com anomalia de Ebstein, incluindo deslocamento apical do folheto septal da valva tricúspide (acolamento de 36,3 mm), valva tricúspide displásica e dilatação discreta do átrio direito, atribuída à atrialização do ventrículo direito. Os septos interatrial e interventricular encontravam-se íntegros, e a fração de ejeção do ventrículo esquerdo estava preservada. A ressonância magnética cardíaca não revelou outras anormalidades estruturais ou funcionais. Considerando a complexidade fenotípica, foi realizado sequenciamento completo do exoma na filha, com o intuito de identificar outras variantes potencialmente relevantes. No entanto, o exame confirmou apenas a presença da variante p.Thr75Met no gene *KCNJ2*, previamente identificada no caso índice. O filho do paciente, por sua vez, não apresentava dismorfias nem alterações em exames cardiológicos realizados. Diante do baixo índice de suspeição clínica, optou-se por investigação molecular dirigida apenas para a variante identificada, por meio de sequenciamento do gene *KCNJ2* (Sanger), com resultado negativo.

Foi instituído tratamento com propranolol para o paciente e sua filha, com melhora observada no prolongamento do intervalo QT em ambos. O paciente também iniciou uso de espironolactona e suplementação oral de potássio (K^+^), com alívio parcial dos sintomas. Orientou-se a ambos a evitar medicamentos conhecidos por prolongar o intervalo QT, conforme listados na base de dados CredibleMeds. Recomendou-se ainda que o irmão do paciente, que apresenta episódios de paralisia periódica, bem como os demais familiares de primeiro grau do caso índice, comparecessem ao ambulatório para realização de teste genético específico e subsequente aconselhamento genético.

Os canais Kir têm estrutura tetramérica com 4 subunidades idênticas ou homólogas formando o poro central que permite a passagem seletiva de íons de potássio. Cada subunidade possui 2 hélices transmembrana (M1 e M2) e um loop H5 que atua como filtro de seletividade, enquanto as extremidades N-terminal e C-terminal regulam a atividade via interação com moléculas como PIP2. Variantes patogênicas no gene *KCNJ2* (a subunidade alfa do canal Kir2.1) causam, entre outros quadros, a SAT, uma canalopatia rara autossômica dominante com paralisia periódica, arritmias ventriculares e dismorfias faciais, decorrente de disfunção na repolarização celular.^1^ A variante p.Thr75Met localiza-se no domínio N-terminal citoplasmático, uma região crucial para a regulação da abertura do canal e seu tráfego para a membrana plasmática. Estudos funcionais demonstram que essa variante compromete o transporte do canal Kir2.1 à membrana plasmática e reduz as correntes de potássio, resultando em perda de função do canal e alterações eletrofisiológicas graves, principalmente pela diminuição da corrente iônica IK1.^7,8^

Clinicamente, a variante p.Thr75Met manifesta-se com expressividade variável, incluindo arritmias ventriculares graves, paralisia periódica e dismorfismos faciais. As arritmias incluem taquicardia ventricular bidirecional, prolongamento do intervalo QTc e ondas U proeminentes, aumentando o risco de *torsades de pointes* e morte súbita cardíaca.^3,6^ A paralisia periódica pode ser normocalêmica ou hipocalêmica, sendo precipitada por jejum, repouso pós-exercício ou ingestão de carboidratos. A penetrância incompleta da variante p.Thr75Met resulta em fenótipos variados, inclusive dentro da mesma família, sugerindo a influência de modificadores genéticos e ambientais. O teste genético é essencial para confirmação diagnóstica e orientação terapêutica individualizada.

Para o manejo das arritmias cardíacas, os betabloqueadores são a primeira linha de tratamento, reduzindo a ectopia ventricular e o risco de arritmias graves. O nadolol é frequentemente preferido devido à sua longa meia-vida e menor variabilidade plasmática. O propranolol também é opção eficaz, especialmente em pacientes que necessitam de betabloqueador mais acessível e amplamente disponível. Já o metoprolol, por ser β1-seletivo, pode ter menor eficácia na prevenção de arritmias ventriculares.^9^ Em casos de arritmias ventriculares refratárias, a fenitoína demonstrou eficácia na sua supressão, possivelmente por seu efeito estabilizador da membrana celular e influência na condução iônica.^10^ Para pacientes de alto risco, o implante de cardiodesfibriladores está indicado para a prevenção primária de morte súbita.^11^ A amiodarona é contraindicada na SAT por prolongar o intervalo QT e aumentar o risco de *torsades de pointes.*^12^

No controle da paralisia periódica associada à síndrome de Andersen-Tawil, a acetazolamida, um inibidor da anidrase carbônica, é frequentemente utilizada; no entanto, a diclorfenamida tem demonstrado superioridade em alguns casos, reduzindo a frequência e a gravidade dos episódios paralisantes.^13^

A anomalia de Ebstein é uma malformação rara da válvula tricúspide, cuja etiologia genética ainda não está completamente elucidada. Variantes no gene *MYH7*, que codifica a cadeia pesada de β-miosina, foram associadas a casos familiares, especialmente quando acompanhadas de não compactação ventricular esquerda.^14,15^ Além disso, microdeleções nos loci 1p36 e 8p23.1, envolvendo genes como *GATA4* e *NKX2-5*, foram identificadas em pacientes com a condição.^16^ Outros estudos indicam que variantes de número de cópias em genes relacionados ao desenvolvimento miocárdico, como *NODAL, PDLIM5, SIX1, ASF1A* e *FGF12*, além de genes da via de sinalização BMP, podem contribuir para sua etiologia.^17,18^ No presente caso, a família apresenta diagnóstico clínico e molecular de SAT causada por uma variante patogênica em *KCNJ2* (p.Thr75Met), e a filha do caso índice apresenta, de forma inédita, a coexistência de anomalia de Ebstein com a SAT — uma associação até o momento não descrita na literatura. Embora não se estabeleça relação causal entre essas condições, a coexistência dos achados levanta a hipótese de influência de fatores genéticos modificadores ou de um espectro fenotípico mais amplo relacionado a *KCNJ2*. Estudos futuros poderão esclarecer se há interações genéticas ou mecanismos compartilhados entre canalopatias e malformações estruturais cardíacas.

Este estudo foi aprovado pelo Comitê de Ética do Hospital Universitário Professor Edgard Santos, da Universidade Federal da Bahia, sob o número de protocolo CAAE 42370821.9.3007.0049. Todos os procedimentos envolvidos neste estudo estão de acordo com a Declaração de Helsinki de 1975, atualizada em 2013. O consentimento informado foi obtido de todos os participantes.
