# Supplementary material for: Variabilidade Fenotípica em uma Família com Síndrome de Andersen-Tawil
Source: Arq Bras Cardiol. 2026 Jan 26;122(12):e20240861. [Article in Portuguese] doi: 10.36660/abc.20240861 (PMC12978374; doi:10.36660/abc.20240861)
Supplement: Translation

# Translation: Phenotypic Variability in a Family with Andersen-Tawil Syndrome

**DOI:** 10.36660/abc.20240861i

**Authors:** Samuel Ulisses Chaves Nogueira do Nascimento, Jussara de Oliveira Pinheiro Duarte, Luiz Pereira de Magalhães, Alex Teixeira Guabiru, Adimeia Souza Santos, Enderson Cavalvanti, Victoria Bastos Rodrigues, Roque Aras

**Affiliations:** 1 Brazil Hospital Universitário Professor Edgard Santos, Salvador, BA – Brazil; 2 Brazil Universidade Federal da Bahia, Salvador, BA – Brazil

**Keywords:** Andersen Syndrome, Paralysis, Cardiac Arrhythmias, Channelopathies, Human Genetics

## Introduction

Cardiac channelopathies are genetic disorders caused by pathogenic variants in genes encoding ion channels, resulting in alterations in cardiac electrophysiology and a predisposition to potentially fatal arrhythmias. Among these conditions, Long QT Syndrome (LQTS) stands out as a heterogeneous clinical entity, characterized by prolonged QT interval and an increased risk of ventricular tachyarrhythmia and sudden death.

Some hereditary forms of LQTS occur in the context of genetic syndromes, such as Andersen-Tawil Syndrome (ATS), which is caused by variants in the KCNJ2 gene, encoding the Kir2.1 potassium channel. In addition to long QT and a predisposition to ventricular arrhythmia, ATS manifests with periodic paralysis and craniofacial dysmorphisms, forming a diagnostic triad. Although the diagnosis may be clinical, molecular confirmation is essential for risk stratification, genetic counseling, and differentiation from other conditions with phenotypic overlap.^1^

This report describes a family with ATS associated with the *KCNJ2:p.Thr75Met* variant, highlighting variable expressivity and an unusual phenotypic finding in the daughter of the index case: Ebstein’s anomaly, which is not associated with the syndrome. The correlation between clinical, molecular, and functional findings emphasizes the relevance of this case for understanding ATS and its atypical manifestations.

## Description

The index case, a 52-year-old man, was referred to our team due to periodic paralysis attacks since age 14, characterized by loss of voluntary movement in the lower limbs, lasting 3 to 5 days. At age 25, the attacks intensified, also affecting the upper limbs, and were triggered by post-exercise rest, carbohydrate intake, and corticosteroids. He reported sporadic palpitations associated with stress. His sister died suddenly at age 37, with possible cardiac involvement related to ATS; a brother has periodic paralysis triggered by exercise.

Physical examination of the index case revealed micrognathia, low-set ears, down-slanting palpebral fissure, high-arched palate, clinodactyly of the fifth finger, and syndactyly of the second and third toes (Figure 1). The electrocardiogram (ECG) showed prolonged QT interval, sinus bradycardia, leftward QRS axis deviation, and incomplete right bundle branch block. A 24-hour Holter monitor identified ventricular bigeminy; the echocardiogram confirmed preserved ejection fraction (79%), with no significant structural changes. Whole-exome sequencing identified the c.224C>T variant (dbSNP: rs104894585), resulting in p.Thr75Met in the *KCNJ2* gene (NM_000891.3) in heterozygosity. The classification as pathogenic followed the ACMG criteria:^2^ in silico predictions (PP3), case-control studies (PS4), functional assays demonstrating loss of Kir2.1 channel function (PS3), low population frequency (PM2), familial segregation (PP1), and previous reports in the literature (PS1).^3-6^ The pedigree (Figure 2) confirmed the autosomal dominant inheritance pattern.

**Figure 1:** – Physical examination revealed micrognathia, low-set ears, downward-slanting palpebral fissure, high-arched palate, bilateral clinodactyly of the fifth finger, and syndactyly of the second and third toes.

**Figure 2:** – Pedigree showing the autosomal dominant pattern of Andersen-Tawil Syndrome (ATS). Patients in red have a clinical diagnosis of ATS. Patients who underwent genetic testing and received confirmation are indicated with “+”. Other family members are still awaiting genetic testing, despite having a clinical diagnosis.

The patient’s children were evaluated to investigate the presence of the variant identified in the index case and possible associated clinical manifestations. The daughter presented dysmorphisms similar to her father’s but did not report episodes of periodic paralysis. Her electrocardiogram revealed marked prolongation of the corrected QT interval (QTc = 579 ms). A 24-hour Holter monitor demonstrated notable ventricular ectopic activity, including episodes of bigeminy, predominantly polymorphic ventricular extrasystoles, as well as nonsustained ventricular tachycardia with instances of polymorphic morphology. Exercise stress testing confirmed the presence of polymorphic ventricular arrhythmia triggered by physical exertion. Transthoracic echocardiography revealed findings consistent with Ebstein’s anomaly, including apical displacement of the tricuspid valve septal leaflet (36.3 mm attachment), a dysplastic tricuspid valve, and mild right atrial dilation attributed to right ventricular atrialization. The interatrial and interventricular septa were intact, and the left ventricular ejection fraction was preserved. Cardiac magnetic resonance imaging revealed no other structural or functional abnormalities. Given the phenotypic complexity, whole-exome sequencing was performed on the daughter to identify other potentially relevant variants. However, the examination only confirmed the presence of the p.Thr75Met variant in the *KCNJ2* gene, previously identified in the index case. The patient’s son, in turn, presented no dysmorphic findings or abnormalities on cardiological examinations. Given the low index of clinical suspicion, a molecular investigation was chosen, targeting only the identified variant, through sequencing of the *KCNJ2* gene (Sanger), with a negative result.

Treatment with propranolol was instituted for the patient and his daughter, with improvement observed in QT interval prolongation in both. The patient also started taking spironolactone and oral potassium supplementation (K⁺), with partial relief of symptoms. Both patients were advised to avoid medications known to prolong the QT interval, as listed in the CredibleMeds database. It was also recommended that the patient’s brother, who experiences episodes of periodic paralysis, as well as other first-degree relatives of the index case, attend the outpatient clinic for specific genetic testing and subsequent genetic counseling.

Kir channels have a tetrameric structure with four identical or homologous subunits forming the central pore that allows the selective passage of potassium ions. Each subunit has two transmembrane helices (M1 and M2) and an H5 loop that acts as a selectivity filter, while the N-terminal and C-terminal ends regulate activity via interactions with molecules such as PIP2. Pathogenic variants in the *KCNJ2* gene (the alpha subunit of the Kir2.1 channel) cause, among other conditions, ATS, a rare autosomal dominant channelopathy with periodic paralysis, ventricular arrhythmias, and facial dysmorphisms, resulting from impaired cellular repolarization.^1^ The p.Thr75Met variant is located in the N-terminal cytoplasmic domain, a region crucial for regulating channel opening and its trafficking to the plasma membrane. Functional studies show that this variant hinders Kir2.1 channel transport to the plasma membrane, lowers potassium currents, and causes significant electrophysiological changes, primarily due to reduced IK1^7,8^ ionic current.

Clinically, the p.Thr75Met variant manifests with variable severity, including severe ventricular arrhythmias, periodic paralysis, and facial dysmorphisms. Arrhythmias include bidirectional ventricular tachycardia, prolonged QTc interval, and prominent U waves, increasing the risk of *torsades de pointes* and sudden cardiac death.^3,6^ Periodic paralyses can be normokalemic or hypokalemic, precipitated by fasting, post-exercise rest, or carbohydrate ingestion. The incomplete penetrance of the p.Thr75Met variant results in varied phenotypes, even within families, suggesting the influence of genetic and environmental modifiers. Genetic testing is essential for diagnostic confirmation and individualized therapeutic guidance.

For the management of cardiac arrhythmias, beta-blockers are the first-line treatment, reducing ventricular ectopy and the risk of serious arrhythmia. Nadolol is often preferred due to its long half-life and lower plasma variability. Propranolol is also an effective option, especially in patients requiring a more affordable and widely available beta-blocker. Metoprolol, being β1-selective, may be less effective in preventing ventricular arrhythmias.^9^ In cases of refractory ventricular arrhythmias, phenytoin is effective in suppressing them, possibly due to its stabilizing effect on the cell membrane and its influence on ionic conduction.^10^ For high-risk patients, implantation of cardioverter-defibrillators is indicated for the primary prevention of sudden death.^11^ Amiodarone is contraindicated in ATS because it prolongs the QT interval and increases the risk of *torsades de pointes.*^12^

In the control of periodic paralysis associated with ATS, acetazolamide, a carbonic anhydrase inhibitor, is frequently used; however, dichlorphenamide has demonstrated superiority in some cases, reducing the frequency and severity of paralytic episodes.^13^

Ebstein’s anomaly is a rare tricuspid valve malformation whose genetic etiology is not yet fully understood. Variants in the MYH7 gene, which encodes the β-myosin heavy chain, have been associated with familial cases, especially when accompanied by left ventricular noncompaction.^14,15^ Furthermore, microdeletions at loci 1p36 and 8p23.1, involving genes such as GATA4 and NKX2-5, have been identified in patients with the condition.^16^ Other studies indicate that copy number variants in genes related to myocardial development, such as NODAL, PDLIM5, SIX1, ASF1A, and FGF12, as well as genes in the BMP signaling pathway, may contribute to its etiology.^17,18^ In this case, the family presents a clinical and molecular diagnosis of ATS caused by a pathogenic variant in KCNJ2 (p.Thr75Met), and the daughter of the index case presents, for the first time, the coexistence of Ebstein anomaly with ATS, an association not previously described in the literature. Although a causal relationship between these conditions has not been established, the coexistence of these findings raises the hypothesis of the influence of modifying genetic factors or a broader phenotypic spectrum related to KCNJ2. Future studies may clarify whether there are genetic interactions or shared mechanisms between channelopathies and cardiac structural malformations.

This study was approved by the Ethics Committee of Professor Edgard Santos University Hospital, Federal University of Bahia, under protocol number CAAE 42370821.9.3007.0049. All procedures involved in this study are in accordance with the 1975 Declaration of Helsinki, updated in 2013. Informed consent was obtained from all participants.
